# DUAL SENSORY IMPAIRMENT AND PHYSICAL FUNCTION AMONG MEXICAN AMERICAN OLDER ADULTS

**DOI:** 10.1093/geroni/igae098.3953

**Published:** 2024-12-31

**Authors:** Arati Bendapudi, Soham Al Snih

**Affiliations:** University of Texas Medical Branch, Galveston, Texas, United States; University of Texas Medical Branch in Galveston, Galveston, Texas, United States

## Abstract

The study objective was to examine the relationship between hearing impairment (HI) and vision impairment (VI) on physical function over 12-years of follow-up among Mexican Americans aged 75 years and older. Participants (N=662) were from the Hispanic Established Population for the Epidemiological Study of the Elderly who scored 7 or more on the Short Physical Performance Battery (SPPB) at baseline. Measures included socio-demographics, medical conditions, pain, depressive symptoms, falls, cognitive function, disability, and handgrip strength. Dual Sensory Impairment (DSI) was defined as the presence of sensory and visual impairment. Physical function impairment (PFI) was defined as having a score of less than 7 on the SPPB. Participants were grouped into four groups: No HI - No VI (n=503), HI only (n=70), VI only (n=32), and VI-HI (n=17). Generalized Estimating Equation models estimated the odds ratio (OR) and 95% Confidence Interval (CI) of PFI as a function of DSI over time after controlling for all covariates. Participants with HI only, and those with DSI had greater odds (OR=1.64, 95% CI=1.17-2.31, p-value=0.0043 and OR=2.35, 95% CI=1.38-4.83, p-value=0.0209, respectively) of developing PFI over time compared to those without DSI after controlling for all covariates. No significant association was found between those with VI only and PFI. In conclusion, Mexican American older adults with DSI were at higher risk of developing PFI over time. These findings highlight the importance of culturally informed clinical practices that address DSI in an underserved population with language barriers and limited access to health care.

